# Geriatric hospitalizations in fall-related injuries

**DOI:** 10.1186/s13049-014-0063-1

**Published:** 2014-11-12

**Authors:** Cheng-Shyuan Rau, Tsan-Shiun Lin, Shao-Chun Wu, Johnson Chia-Shen Yang, Shiun-Yuan Hsu, Tzu-Yu Cho, Ching-Hua Hsieh

**Affiliations:** Department of Neurourgery, Kaohsiung Chang Gung Memorial Hospital and Chang Gung University College of Medicine, Kaohsiung City, Taiwan; Department of Trauma Surgery, Kaohsiung Chang Gung Memorial Hospital and Chang Gung University College of Medicine, No.123, Ta-Pei Road, Niao-Song District, Kaohsiung City, 833 Taiwan; Department of Anesthesiology, Kaohsiung Chang Gung Memorial Hospital and Chang Gung University College of Medicine, Kaohsiung City, Taiwan; Department of Plastic and Reconstructive Surgery, Kaohsiung Chang Gung Memorial Hospital and Chang Gung University College of Medicine, Kaohsiung City, Taiwan

**Keywords:** Elderly, Injury Severity Score (ISS), Mortality, Fall

## Abstract

**Background:**

To investigate the injury pattern, severity, and mortality of elderly patients hospitalized for treatment of trauma following fall accidents.

**Methods:**

Data obtained from the Trauma Registry System were retrospectively reviewed for trauma admissions between January 1, 2009 and December 31, 2013 in a Level I trauma center. Of 16,548 registered patients, detailed information was retrieved from the 2,403 elderly patients (aged 65 years and above) with fall accidents and was compared with information from 1,909 adult patients (aged 20–64) with fall accidents.

**Results:**

Falls presented the major mechanism for admission (59.9%) in the elderly patients. The number of elderly patients who fell from a height <1 m was greater than that of the adult patients (91.9% vs. 62.5%, respectively, p <0.001). The Injury Severity Score (ISS) (9.3 ± 4.4 vs. 8.3 ± 6.1, respectively, p =0.007) and New Injury Severity Score (NISS) (10.3 ± 6.8 vs. 9.5 ± 8.2, respectively, p <0.001) were significantly higher in the elderly than the adult patients. A significantly larger proportion of the elderly patients were admitted to the ICU (16.2% vs. 13.4%, respectively, p =0.009), and the elderly were found to have longer stays in the intensive care unit (ICU) (8.6 days vs. 7.6 days, respectively, p =0.034) but not in the hospital in general (9.6 days vs. 8.5 days, respectively, p =0.183). Additionally, a significantly higher percentage of the elderly patients sustained subdural hematoma (10.1% vs. 8.2%, respectively, p =0.032) and femoral fracture (50.6% vs. 14.1%, respectively, p <0.001). There were significant differences in in-hospital mortality (18.2% vs. 10.3%, respectively, p =0.031) and length of stay in the hospital (11.6 days vs. 14.9 days, respectively, p =0.037) between the elderly and adult patients with subdural hematoma, but not between those with femoral fracture.

**Conclusions:**

Analysis of the data indicates that elderly patients hospitalized for treatment of trauma following fall accidents present with a bodily injury pattern that differs from that of adult patients and have a higher severe injury score, worse outcome, and higher mortality than those of adult patients.

## Background

Falls are a leading cause of injury and death among the elderly and a significant public health issue [1–3]. Approximately one-third of the population over the age of 65 experiences falls each year, a figure that rises to over 50% among individuals aged 80 and above [4,5]. In addition, the incidence of falls that lead to admission to emergency units is increasing with the increased size and rapid growth of the geriatric population [6,7]. The rates of fall-induced deaths and the absolute number of these deaths, are increasing rapidly [8,9]. Therefore, considering the fact that approximately 25% of the population of Western countries will be in the geriatric age group by the year 2030, hospitalization of geriatric patients for fall-related injuries will become a major issue in the future [10]. Moreover, the acute medical care costs of fall-related injuries will continue to rise in the growing population of the elderly who are living longer and engaging in activities at higher risk of injury [11].

There is strong evidence that elderly trauma patients are at an increased risk of morbidity and mortality compared to younger patients [12–14]. In addition, elderly patients sustain distinct patterns of injuries from causes that differ from those of non-elderly adults because of their unique anatomical, physiologic, and behavioral characteristics. The effect of trauma would decrease both the ability to live an active lifestyle and the physiologic capacity in elderly patients [15]. Geographic variation in trauma patterns may occur, but there is limited information available about falls among the geriatric population in Taiwan. The purpose of this epidemiologic study was to assess the clinical characteristics and outcomes of elderly patients admitted for treatment for fall-related injuries in a Level I trauma center over a five-year period using data from a population-based trauma registry.

## Methods

### Study design

The study was conducted at Kaohsiung Chang Gung Memorial Hospital, a 2,400-bed facility and a Level I regional trauma center that provides care to trauma patients primarily from South Taiwan. Approval for this study was obtained from the hospital’s institutional review board (approval number 103-3035B) before its initiation. This retrospective study was designed to review all the data added to the Trauma Registry System from January 1, 2009 to December 31, 2013 for cases that visited our emergency room with any trauma mechanism and met the inclusion criteria of (1) age ≥65 years and (2) hospitalization for treatment of trauma sustained in a fall. For comparison, data regarding adults aged 20 to 64 years old were also collected.

Among the 16,548 hospitalized registered patients entered in the database, 4,011 (24.2%) were ≥65 years of age (hereafter referred to as the elderly) and 10,234 (61.8%) were 20 to 64 years of age (hereafter referred to as adults). Cut-off age of 20 was arbitrarily selected to be an adult because there is a legal requirement of supervisor to proceed health care such as admission, invasive procedure, and operation in Taiwan until the patient turns 20 years old. Among these patients, 2,403 (59.9%) elderly and 1,909 (18.7%) adults had been admitted due to a fall accident. Detailed patient information was retrieved from the Trauma Registry System of our institution and included data regarding age, sex, height of fall, and vital signs on arrival, as well as the first Glasgow Coma Scale (GCS) score in the emergency department, Abbreviated Injury Scale (AIS) scores for each body region, Injury Severity Score (ISS), New Injury Severity Score (NISS), Trauma and Injury Severity Score (TRISS), length of hospital stay (LOS), length of intensive care unit stay (LICUS), in-hospital mortality, and associated complications. AIS scores every injury and classified each according to six severity as (1) minor, (2) mild, (3) serious, (4) severe, (5) critical, and (6) mortal [16]. The ISS is the sum of the square of the three most severe injuries, but it only considers one injury per body region [17]. The NISS, a modification of the ISS in 1997, is defined as the sum of a patient’s three most severe injuries, regardless of body region [18]. The TRISS utilizes the patient’s age, type of injury, Revised Trauma Score (RTS), and the ISS to estimate the probability of survival [19]. Among the TRISS, the RTS is calculated using the GCS score, the systolic blood pressure (SBP), and respiratory rate (RR) [20]. In brief, AIS, ISS, and NISS are anatomical, GCS and RTS are physiological scoring systems and TRISS is a combined scoring system for survival prediction. In this study, the primary outcome is the injury severity by different scoring system (GCS, AIS, ISS, NISS, TRISS) and the secondary outcome is LOS, LICUS, and in-hospital mortality. In addition, the pre-existed comorbidities and chronic diesases including diabetes mellitus (DM), hypertension (HTN), coronary artery diseases (CAD), congestive heart failure (CHF), cerebrovascular accident (CVA), and end-stage renal disease (ESRD) were identified. Adjusted odd ratios (AOR) of the mortality according to these pre-existed comorbidities, chronic diesases and ISS with 95% confidence intervals (CI) of this AOR calculated. The data collected regarding the populations of elderly and adults were compared using SPSS v.20 statistical software (IBM Corporation, Armonk, NY, USA) for performance of Pearson’s chi-squared test, Fisher’s exact test, or the independent Student’s *t* test, as applicable. All results are presented as the mean ± standard error. A p-value less than 0.05 was considered statistically significant.

## Results

### Patient characteristics

**Figure 1:**
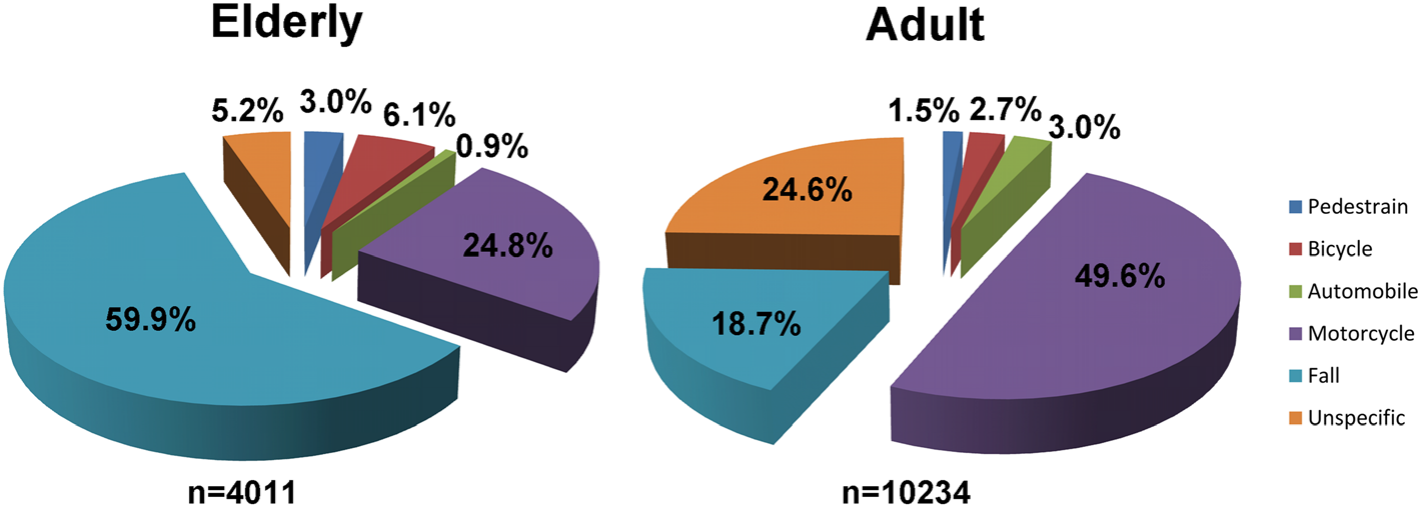
**Etiology of trauma among elderly and adult patients admitted for treatment of trauma injuries.**

As shown in Figure 1, fall presented the major mechanism for admission (59.9%) in the elderly patients, followed by motorcycle accident (24.8%). In contrast, motorcycle accident presented the major mechanism for admission (49.6%) in the adult patients, followed by unspecific mechanisms, which included fight, suicide, and unknown and unclassified injuries. Fall presented the mechanism for admission in 18.7% of the injured adult patients.

Of the 2,403 elderly and 1,909 adult patients injured in a fall, the mean ages were 78.0 ± 7.3 and 49.8 ± 11.1 years, respectively (Table 1). A statistically significant difference regarding sex was found between the elderly. In the group of elderly patients, more than two times as many women as men were injured in a fall; however, among the adult patients, more men were injured in a fall. There were significant higher incidence rates of the pre-existed comorbidities and chronic diesases including DM, HTN, CAD, CHF, CVA, and ESRD in the elderly patients. Analysis of the data regarding height of fall (<1 m, 1–6 m, >6 m) revealed that the majority of both the elderly and adult patients fell from a height <1 m, but more adult than elderly patients fell from a height between 1–6 m and >6 m, implying that the majority of the elderly patients sustained a ground-level fall occurring upon walking or with movement and that more adult than elderly patients sustained a non-ground-level fall occurring with more rigorous activity. No significant difference was found between the elderly and adult patients regarding GCS score or distribution of patients at different levels of consciousness (GCS ≤8, 9–12, or ≥13). Analysis of AIS scores revealed that the elderly patients sustained significantly higher rates of extremity injury than the adult patients, while the adult patients sustained significantly higher rates of injuries to face, thorax, and abdomen. On the other hand, no significant differences regarding injury to the head/neck region were found between the elderly and adult patients.

**Table 1:** **Demographics of hospitalized elderly and adult trauma patients in fall accidents**

**Variable**	**Elderly**		**Adult**		***p***
	**N = 2403**		**N = 1909**		
Age	78.0	±7.3	49.8	±11.1	
Gender, n (%)					<0.001
Male	788	(32.8)	1171	(61.3)	
Female	161	(67.2)	73	(38.7)	
Co-morbidity					
DM	740	(30.8)	295	(15.5)	<0.001
HTN	1493	(62.1)	451	(23.6)	<0.001
CAD	221	(9.2)	48	(2.5)	<0.001
CHF	71	(3.0)	16	(0.8)	<0.001
CVA	335	(13.9)	168	(3.6)	<0.001
ESRD	123	(5.1)	64	(3.4)	0.005
Fall height, n (%)					
<1 m	2209	(91.9)	1194	(62.5)	<0.001
1-6 m	191	(7.9)	672	(35.2)	<0.001
>6 m	3	(0.1)	43	(2.3)	<0.001
GCS	14.5	±1.9	14.4	±2.0	0.283
≤8	69	(2.9)	74	(3.9)	0.067
9-12	92	(3.8)	57	(3.0)	0.132
≥13	2242	(93.3)	1778	(93.1)	0.833
AIS, n (%)					
Head/Neck	510	(21.2)	451	(23.6)	0.060
Face	99	(4.1)	161	(8.4)	<0.001
Thorax	129	(5.4)	209	(10.9)	<0.001
Abdomen	102	(4.2)	149	(7.8)	<0.001
Extremity	1857	(77.3)	1408	(73.8)	0.007
ISS, n (%)					
<16	2106	(87.6)	1646	(86.2)	0.169
16-24	229	(9.5)	195	(10.2)	0.453
≥25	68	(2.8)	68	(3.6)	0.172
ISS	9.3	±4.4	8.3	±6.1	<0.001
<16	8.0	±2.4	6.4	±2.9	<0.001
16-24	16.7	±1.4	17.3	±2.1	<0.001
≥25	25.8	±2.2	29.4	±8.0	<0.001
NISS	10.3	±6.8	9.5	±8.2	<0.001
TRISS	0.99	±0.12	0.99	±0.12	0.768
Mortality, n (%)	78	(3.2)	25	(1.3)	<0.001
Men	46	(5.8)	22	(1.9)	
Female	32	(2.0)	3	(0.4)	
AOR (95%CI)	0.40	(0.13-1.20)			0.103
LOS (days)	9.6	±8.7	8.5	±9.4	0.183
ICU					
Patients, n (%)	390	(16.2)	225	(13.4)	0.009
<16	175	(8.3)	85	(5.2)	<0.001
16-24	153	(66.8)	115	(59.0)	0.095
≥25	62	(91.2)	55	(80.9)	0.083
LICUS (days)	8.6	±11.4	7.6	±9.1	0.034
<16	6.8	±8.3	5.1	±5.8	0.032
16-24	8.8	±12.3	7.4	±10.0	0.092
≥25	13.3	±14.7	12.0	±9.6	0.018

Comparison of trauma injury scores for the elderly and adult patients indicated significant differences regarding ISS (9.3 ± 4.4 vs. 8.3 ± 6.1, respectively, p <0.001). The ISS was higher in the elderly than that in the adult patients; however, when stratified into different groups of injury severity (<16, 16–24, ≥25), the ISS of the elderly was higher than that of the adult patients only in the group of ISS <16. In the groups of ISS between 16-24 and ≥25, the ISS of the adult patients was significantly higher than that of the elderly. Likewise, there were also significant differences regarding NISS and in-hospital mortality, but not TRISS between these two groups of patients. The mortality rate was higher among men than women in both the elderly and the adult patients. However, there was no significant different AOR of the mortality for the elderly after the adjustment according to the pre-existing comorbidities, chronic diseases, and ISS (AOR =0.40, 95% CI: 0.13-1.20), indicating the pre-existing comorbidities and chronic disease may be responsible for the higher incidence rates of mortality in the elderly. No significant differences were found between the elderly and adult patients regarding hospital LOS. However, a significantly larger proportion of the elderly patients were admitted to the ICU, with longer LICUS. Among patients with ISS <16, 8.3% of the elderly and 5.2% of the adult patients were admitted to the ICU (p <0.001), with longer LICUS. In addition, 66.8% of the elderly and 59.0% of the adult patients with ISS 16–24, and 91.2% of the elderly and 80.9% of the adult patients with ISS ≥25 were admitted to the ICU. No significant differences regarding the proportion of patients admitted to the ICU and LICUS were found between the elderly and adult patients with ISS 16–24 and ISS ≥25. However, among patients admitted to ICU and with ISS ≥25, the elderly experienced longer stays in the ICU than the adult patients.

Table 2 shows the findings regarding injury associated with the fall accidents. As observed, a significantly higher percentage of elderly than adult patients sustained subdural hematoma and femoral fracture, but the elderly experienced a significantly lower percentage of other injuries. Furthermore, subsequent analysis focused on subdural hematoma (Table 3) and femoral fracture (Table 4), the associated injuries, which were significantly higher among the elderly with fall accidents. In the patients with subdural hematoma following a fall (elderly n =242, adults n =156), the analysis revealed significant differences in sex between the elderly. No significant difference was found between the elderly and adult patients with subdural hematoma regarding GCS score. The ISS was higher in the adult patients than that in the elderly (18.8 ± 7.8 vs. 17.1 ± 5.7, respectively, p <0.001). In addition, there were significant differences between the elderly and the adult patients with subdural hematoma regarding in-hospital mortality and length of stay in the hospital. Even after the adjustment according to the pre-existing comorbidities, chronic diseases, and ISS, there was still a greater odds of mortatlity for the elderly (AOR =1.9, 95% CI: 1.06-3.59). However, the mortality rate among men and women with subdural hematoma was not significantly different in either the elderly or the adult patients. No significant differences were found between the elderly and adult patients with subdural hematoma regarding NISS, TRISS, proportion of patients admitted to the ICU and receiving operations, and the number of operations. However, among the patients with ISS <16, the elderly had a significantly longer length of stay in the hospital than the adult patients.

**Table 2:** **Associated injuries of hospitalized elderly and adult patients in fall accidents**

**Fall accident**					
**Variable**	**Elderly**		**Adult**		***p***
	**N = 2403**		**N = 1909**		
Head trauma, n (%)					
Neurologic deficit+	1	(0.04)	10	(0.5)	0.002
Cranial fracture+	51	(2.1)	81	(4.2)	<0.001
Epidural hematoma (EDH)+	36	(1.5)	60	(3.1)	<0.001
Subdural hematoma (SDH)*	242	(10.1)	156	(8.2)	0.032
Subarachnoid hemorrhage (SAH)	134	(5.6)	128	(6.7)	0.123
Intracerebral hematoma (ICH)	49	(2.0)	28	(1.5)	0.159
Cerebral contusion	104	(4.3)	95	(5.0)	0.313
Cervical vertebral fracture+	16	(0.7)	29	(1.5)	0.006
Maxillofacial trauma, n (%)					
Maxillary fracture+	20	(0.8)	47	(2.5)	<0.001
Mandibular fracture+	2	(0.1)	20	(1.0)	<0.001
Orbital fracture+	5	(0.2)	12	(0.6)	0.029
Nasal fracture	6	(0.2)	10	(0.5)	0.141
Thoracic trauma, n (%)					
Rib fracture+	77	(3.2)	142	(7.4)	<0.001
Sternal fracture+	0	(0.0)	5	(0.3)	0.012
Hemothorax+	14	(0.6)	25	(1.3)	0.012
Pneumothorax+	5	(0.2)	31	(1.6)	<0.001
Lung contusion+	2	(0.1)	12	(0.6)	0.002
Hemopneumothorax+	5	(0.2)	31	(1.6)	<0.001
Thoracic vertebral fracture+	37	(1.5)	57	(3.0)	0.001
Abdominal trauma, n (%)					
Intra-abdominal injury+	3	(0.1)	20	(1.0)	<0.001
Hepatic injury+	3	(0.1)	18	(0.9)	<0.001
Splenic injury	1	(0.04)	5	(0.3)	0.054
Retroperitoneal injury	2	(0.1)	4	(0.2)	0.269
Renal injury	3	(0.1)	7	(0.4)	0.101
Urinary bladder injury	1	(0.04)	4	(0.2)	0.108
Lumbar vertebral fracture+	64	(2.7)	112	(5.9)	<0.001
Sacral vertebral fracture+	4	(0.2)	22	(1.2)	<0.001
Extremity trauma, n (%)					
Scapular fracture	6	(0.2)	10	(0.5)	0.141
Clavicle fracture+	25	(1.0)	43	(2.3)	0.002
Humeral fracture	118	(4.9)	101	(5.3)	0.572
Radial fracture+	224	(9.3)	363	(19.0)	<0.001
Ulnar fracture+	100	(4.2)	119	(6.2)	0.002
Femoral fracture*	1215	(50.6)	270	(14.1)	<0.001
Patella fracture+	55	(2.3)	100	(5.2)	<0.001
Tibia fracture+	44	(1.8)	111	(5.8)	<0.001
Fibular fracture+	26	(1.1)	67	(3.5)	<0.001
Metacarpal fracture+	7	(0.3)	35	(1.8)	<0.001
Metatarsal fracture+	12	(0.5)	135	(7.1)	<0.001
Calcaneal fracture+	57	(2.4)	135	(7.1)	<0.001
Pelvic fracture+	25	(1.0)	58	(3.0)	<0.001

+ and * indicated significant lower and higher incidences of the associated injury, respectively, in the elderly than those adult patients ( p<0.05). DM: diabetes mellitus; HTN: hypertension; CAD: coronary artery diseases; CHF: congestive heart failure; CVA: cerebrovascular accident; ESRD: end-stage renal disease.

**Table 3:** **Injury characteristics of hospitalized elderly and adult patients with subdural hematoma in fall accidents**

**Subdural hematoma**					
**Variables**	**Elderly**		**Adult**		***p***
	**N = 242**		**N = 156**		
Age	77.7	±7.6	49.4	±11.0	
Gender, n (%)					<0.001
Male	130	(53.7)	125	(80.1)	
Female	112	(46.3)	31	(19.9)	
GCS	12.3	±3.9	11.9	±4.3	0.341
ISS	17.1	±5.7	18.8	±7.8	<0.001
NISS	21.6	±12.4	24.0	±14.6	0.274
TRISS	0.849	±0.192	0.885	±0.181	0.261
Mortality, n (%)	44	(18.2)	16	(10.3)	0.031
Male	27	(20.8)	13	(10.4)	
Female	17	(15.2)	3	(9.7)	
AOR (95% CI)	1.9	(1.06-3.59)			0.033
LOS (days)	11.6	±11.6	14.9	±14.0	0.037
ICU					
Patients, n (%)	173	(71.5)	106	(67.9)	0.452
<16	20	(48.8)	13	(54.2)	0.675
16-24	107	(71.3)	59	(65.6)	0.348
≥25	46	(90.2)	34	(81.0)	0.201
LICUS (days)	8.5	±10.7	7.8	±8.5	0.158
<16	5.6	±6.1	3.0	±0.8	0.014
16-24	7.9	±10.	6.6	±8.2	0.130
≥25	11.1	±13.1	11.7	±9.2	0.119
Operation, n (%)					0.102
Yes	58	(24.0)	49	(31.4)	
No	184	(76.0)	107	(68.6)	
Operation	1.4	±0.7	1.6	±1.0	0.063

**Table 4:** **Injury characteristics of hospitalized elderly and adult patients with femoral fracture in fall accidents**

**Femoral fracture**					
**Variables**	**Elderly**		**Adult**		***p***
	**N = 1215**		**N = 270**		
Age	79.5	±7.0	53.8	±9.5	
Gender, n (%)					<0.001
Male	336	(27.7)	147	(54.4)	
Female	879	(72.3)	123	(45.6)	
ISS	9.2	±1.5	9.7	±3.2	<0.001
NISS	9.6	±2.1	10.1	±3.7	<0.001
TRISS	0.965	±0.013	0.975	±0.019	<0.001
Mortality, n (%)	14	(1.2)	1	(0.4)	0.245
Male	5	(1.5)	1	(0.7)	
Female	9	(1.0)	0	(0.0)	
AOR (95%CI)	3.1	(0.41-23.95)			0.271
LOS (days)	9.8	±7.1	8.5	±7.3	0.846
ICU					
Patients, n (%)	84	(6.9)	12	(4.4)	0.136
<16	78	(6.5)	7	(2.7)	0.017
16-24	4	(44.4)	1	(20.0)	0.360
≥25	2	(40.0)	4	(100.0)	0.058
LICUS (days)					
<16	6.2	±9.2	7.9	±4.3	0.733
16-24	12.5	±12.8	4		-
≥25	16.5	±13.4	6.5	±3.7	0.003
Operation, n (%)					<0.001
Yes	438	(36.0)	142	(52.6)	
No	777	(64.0)	128	(47.4)	
Operation	1.0	±0.8	1.0	±0.8	0.962
Diagnosis					
Femoral head	43	(3.5)	24	(8.9)	<0.001
Femoral neck	533	(43.9)	112	(41.5)	0.474
Intertrochanteric	586	(48.2)	89	(33.0)	<0.001
Femoral shaft	70	(5.8)	53	(19.6)	<0.001

In the patients with femoral fracture following a fall (elderly n =1,215, adults n =270), significant differences regarding sex were found between the elderly. The adult patients had significantly higher ISS (9.7 ± 3.2 vs. 9.2 ± 1.5, respectively, p <0.001), NISS, and TRISS than the elderly. However, there were no significant differences between the elderly and the adult patients with femoral fracture regarding in-hospital mortality, adjusted odds of mortality (AOR =3.1, 95% CI: 0.41-23.95) and length of stay in the hospital. The mortality rate among men and women with femoral fracture was also not significantly different in either the elderly or the adult patients. There was a significantly higher proportion of patients admitted to the ICU among the group of elderly patients with ISS <16 when compared to the adult patients in the same group of injury severity; however, the length of stay in the ICU did not differ between these two groups of patients with femoral fracture. In contrast, elderly patients with ISS ≥25 experienced longer stays in the ICU than adult patients with ISS ≥25. The proportion of adult patients receiving operations was significantly higher than that of the elderly, but the number of operations performed did not differ. Regarding the location of the femur fracture, the elderly sustained more intertrochanteric fracture but less femoral shaft fracture and femoral head fracture than the adult patients.

## Discussion

This study analyzed the demographics and characteristics of injuries observed in a geriatric population presenting at a Level I trauma center following fall accidents. Analysis of the data indicates that elderly patients present with a bodily injury pattern that differs from that of adult patients, and have a higher injury severity, worse outcome, and higher mortality than those of adult patients.

Notably, in this study, the elderly patients had a mean age of close to 80, much higher than the cutoff of 65 used in the general part of this study, and represent a even higher risk group. As shown, more than two times as many elderly women as men were injured in a fall; however, greater mortality was noted among the elderly men than among the elderly women. In contrast, among the adult patients, more men were injured, and the men sustained a higher fatality rate in the fall accidents than that of the women. These results are in agreement with those of studies showing that falls are more common in older women than men, that elderly women account for the majority of fall-related emergency department visits [2,21,22], and that the death rate associated with falls is 46% higher for men than for women [23]. Although some studies have found that men comprised a significantly greater proportion of fall victims [24,25], and the exact reasons for the disparity between men and women in fall-induced deaths are still largely unknown [26], elderly men might engage in riskier behavior and then perhaps sustain more severe injuries that required an emergency department visit and subsequent hospitalization [24]. In the present study, high-energy falls were less common among the elderly than in the adult population. The majority of the elderly sustained a ground-level fall was supposed to occur upon walking or with movement, and more adult patients sustained a non-ground-level fall occurring with more rigorous activity. This observation may contribute to the discrepancy in the proportion of patients and fatality rates according to gender among elderly and adult patients in fall accidents. Variations in lifestyle habits between people in different regions may also affect the different patterns of trauma in a fall [25]. However, our registered data were unable to provide more detailed information regarding the activity that induced the fall in the current study.

The factors affecting mortality in falls are very complex [27]. The mortality rates associated with falls from 3–6 m, 6–10 m, and >10 m were found to be increased 5, 6.5, and 13 times, respectively, in comparison with the mortality rates of falls from 3 m or less [27]. In the present study, although more adult than elderly patients fell from a height between 1–6 m and >6 m, the mortality rates associated with falls were higher in the elderly than the younger population. (3.2% vs. 1.3%, respectively, p <0.001). Beside the age of the patient and the height of the fall, suicide attempt, type of ground on which the patient fell, place of fall, and head, thoracic, and abdominal trauma are the primary factors affecting mortality [27]. In addition, associated predisposing comorbidity is another factor that may influence the outcome in fall accidents. This current study revealed that these patients don’t do worse because they are older, they do worse because they are chronically sicker. Brain injuries and injuries to the lower extremity are the most fatal of fall injuries in the elderly, accounting for 78% of fatalities and 79% of fall-related costs [28]. Deaths due to falls result from multiple blunt traumas, especially head trauma [29]. In a postmortem study of 484 patients who had fallen or jumped from a height, the most frequently affected body part was the head (91%) [30], followed by the thorax (54%), abdomen (37%), extremities (36%), and neck (17%). Falls are the most common cause of traumatic brain injury in the elderly and account for 46% of all fall-related deaths [1,31]. Among the pathologies related to head trauma, subdural hematoma was found to account for half of the mortality cases [32]. In the present study, the mortality of the elderly with subdural hematoma was found to be 18.2% (44/242), and, of the 78 fatal cases among the elderly patients, there were 44 (56%) fatal cases of subdural hematoma. Notably, this study revealed that TRISS, a combination index developed by the Major Trauma Outcome Study (MTOS) performed in 1982 [33] based on revised trauma score (RTS), ISS, and patient’s age to predict the mortality of injured patients, was not adequate to estimate the fatality rate among the elderly in fall accidents. TRISS appears to be valid for both adult and pediatric trauma cases [34]; however, because TRISS does not take into account pre-existing medical conditions [34], its application to elderly patients in fall accidents should be validated and a modified version of TRISS may need to be developed for better prediction of survival in elderly patients sustaining fall accidents.

As shown in this study, falls are the leading cause of nonfatal injury in the elderly population [35,36]. It had been reported that fractures occur in 3–12% of falls in the elderly and that hip fractures occur in less than 1% of all falls [37,38]; however, 90% of all hip fractures in the elderly are caused by a fall [39]. Occurring in 33% of nonfatal falls, fractures are the most common and costly fall injuries, and account for 61% of nonfatal fall-related costs [28]. In the first year following a femur fracture, 25% of elderly patients will die [40], 50% will experience a decline in performance of activities of daily living (ADLs) [40], and 76% will experience a decline in their motility [1]. In the present study, a remarkably higher percentage of elderly patients suffered femoral fracture compared to the adult patients (50.6% vs. 14.1%, respectively, p <0.001), and hip fracture, a femoral fracture that occurs in the proximal end of the femur, comprised 96.5% of femoral fracture in the elderly patients in fall accidents. In addition, the majority of elderly patients with a femoral fracture did not undergo surgery. The reasons, yet unidentified, may be attributed to a different injury pattern or conservative altitude of surgeons to perform operation in the elderly. In Taiwan, hip fracture rates are among the highest in the world, and the age-specific incidence rates of hip fractures have been found to increase with increasing age in both genders, in an exponential manner after 65 years of age [41]. The overall incidence of hip fractures showed a significant 30% increase (p <0.0001) from 1996 to 2002, from 49.56 to 64.37 per 10,000 per year [42]. Notably, however, from 1999 to 2010, there was a decline in hip fracture rates among elderly Taiwanese adults with a concomitant increase in anti-osteoporosis medication expenditure [43]. Therefore, development and implementation of public health strategies for fall prevention should focus more on this geriatric group in Taiwan’s rapidly aging society.

Our findings must be considered with some cautions. The limitations of this study include the use of a retrospective design and the lack of availability of data regarding the reasons, the risk factors, and circumstances of the mechanism of injury. Additionally, our single center study may not be representative of the entire population of Taiwan and our results may therefore not be generalizable. It is also possible that some of our patients suffered during the study period from recurrent falls that did not require emergency department visits and subsequent admissions, or following which these patients were treated elsewhere. In addition, the impact of pre-existing comorbidities in the elderly on the hospitalization course and on the mortality remains unclarified.

## Conclusion

Elderly patients in fall accidents tend to experience a higher injury severity, a worse outcome, and a higher mortality rate compared to adult patients, as well as a bodily injury pattern differing from that of adults, indicating the need to emphasize fall prevention to reduce both the rate of falls and the associated injuries.
